# Platelet-derived growth factor-BB regenerates functional periodontal ligament in the tooth replantation

**DOI:** 10.1038/s41598-022-06865-6

**Published:** 2022-02-25

**Authors:** Koichiro Komatsu, Hisashi Ideno, Tatsuya Shibata, Kazuhisa Nakashima, Akira Nifuji

**Affiliations:** 1 Department of Pharmacology, School of Dental Medicine, Tsurumi University, 2-1-3 Tsurumi, Tsurumi-ku, Yokohama, 230-8501 Japan; 2 Division of Dental Pharmacology, Department of Oral Science, School of Dentistry, Oh-U University, 31-1 aza Sankakudoh, Tomita-machi, Kohriyama, 963-8611 Japan

**Keywords:** Experimental models of disease, Musculoskeletal system, Ligaments, Anatomy, Biomedical engineering

## Abstract

Tooth ankylosis is a pathological condition of periodontal ligament (PDL) restoration after tooth replantation. Platelet-derived growth factor-BB (PDGF-BB) has been proposed as a promising factor for preventing tooth ankylosis. Using rat tooth replantation model, we investigated whether PDGF-BB accelerates the repair of PDL after tooth replantation without ankylosis, and its molecular mechanisms. In PDGF-BB pretreated replanted teeth (PDGF-BB group), ankylosis was markedly reduced and functionally organized PDL collagen fibers were restored; the mechanical strength of the healing PDL was restored to an average of 76% of that in non-replanted normal teeth at 21 days. The numbers of PDGF-Rβ- and BrdU-positive cells in the periodontal tissues of the PDGF-BB group were greater than those of atelocollagen pretreated replanted teeth (AC group). Moreover, in the PDGF-BB group, the periodontal tissues had fewer osteocalcin-positive cells and decreased number of nuclear β-catenin-positive cells compared to those in the AC group. In vitro analyses showed that PDGF-BB increased the proliferation and migration of human periodontal fibroblasts. PDGF-BB downregulated mRNA expressions of RUNX2 and ALP, and inhibited upregulatory effects of Wnt3a on β-catenin, AXIN2, RUNX2, COL1A1, and ALP mRNA expressions. These findings indicate that in tooth replantation, topical PDGF-BB treatment enhances cell proliferation and migration, and inhibits canonical Wnt signaling activation in bone-tooth ankylosis, leading to occlusal loading of the PDL tissues and subsequent functional restoration of the healing PDL. This suggests a possible clinical application of PDGF-BB to reduce ankylosis after tooth replantation and promote proper regeneration of PDL.

## Introduction

Tooth replantation is a clinical procedure for replacing an accidentally or intentionally avulsed tooth in a socket. During the restoration of tooth replantation, periodontal ligament (PDL) is regenerated through the migration and differentiation of PDL stem cells and/or mesenchymal stem cells. One of the clinical problems in tooth replantation is the occurrence of ankylosis, which fuses teeth to the alveolar bone by aberrant differentiation of PDL cells^1,2^. In ankylosis, part of the tooth function is lost due to missing PDL at the ankylosed site. Successful tooth replantation involves repair and regeneration of PDL, which anchors the tooth to bone, provides physiologic mobility, and supports the tooth against occlusal forces.

The tooth replantation animal model represents an important tool for exploring the mechanisms of ankylosis and assessing the effects of various drugs for the prevention of ankylosis^3,4^. A previous study showed that local pretreatment of teeth with platelet-derived growth factor (PDGF-BB) reduced ankyloses in replanted dog teeth^5^. However, whether the application of PDGF-BB restores PDL function and the mechanisms by which PDGF-BB protects against ankyloses remain to be elucidated.

PDGF, the major serum polypeptide growth factor, is stored in platelets and is released during blood clotting^6^. PDGF mediates normal tissue repair processes and stimulates wound healing in soft tissues^6,7^. The application of growth factors, including PDGF-BB, has been shown to stimulate the regeneration of periodontal tissues^8^ in animal experiments^9–11^ and clinical trials^12,13^. Thus, the FDA has approved PDGF-BB for use in periodontal therapy in cases of intrabony defects, furcation lesions, and gingival recessions^8^. PDGF is a potent chemoattractant for neutrophils, monocytes/macrophages, and fibroblasts, and is a potent mitogen for mesenchymal cells such as fibroblasts, PDL cells, and alveolar bone cells^14–16^. Furthermore, PDGF-BB stimulates the expression of type I collagen and collagenase in these cells^15,17^. This suggests that PDGF plays an important role in the initiation and progression of PDL repair in vivo^11^.

Measurements of the biomechanical properties of PDL have been performed to assess the functional repair of the healing PDL after extrusive luxation of monkey teeth^18^, repair of the injured PDL after rat tooth replantation^4^, and the effect of alendronate on the repair of the injured PDL in replanted rat teeth^3^. From a functional point of view, the biomechanical properties of a wound are important for the repair of PDL^19^. However, little is known about how PDGF affects the biomechanical restoration of injured PDL.

Recently, it was reported that aberrantly elevated canonical Wnt signaling in daβcat^Ot^ mice caused dental ankylosis^20^. This study described mineralization of the PDL of molar and incisor teeth, and more stabilized β-catenin positive cells in the periodontal tissues. Therefore, ankylosis occurring in tooth replantation may be caused by aberrant canonical Wnt signaling, and could be affected by PDGF during the PDL repair process.

Thus, the aim of the present study was to examine whether pretreatment of replanted teeth with PDGF-BB effectively reduces ankylosis and restores the mechanical properties of the PDL of the replanted tooth. We further investigated the mechanistic relation between PDGF-BB and Wnt signaling in vivo and in vitro in PDL restoration.

## Results

### PDGF-BB effectively suppressed ankylosis of replanted teeth

We investigated with the effects of PDGF-BB on the restoration of injured periodontal tissues after tooth replantation, and the design of the present study is shown in Supplemental Fig. S1A. We started with macroscopic observation of the replanted teeth using computed tomography (CT) and confocal microscopy. Supplemental Figure S2A shows representative mesio-distal images, reconstructed from CT, of PBS pretreated (PBS), atelocollagen pretreated (AC), and PDGF-BB pretreated (PDGF-BB) replanted teeth 21 days after replantation, and non-replanted (NC) teeth at equivalent age. We noted areas of radio-opacity in CT images that showed ankylosis between the mesial or distal root surfaces and the alveolar bone surfaces in the PBS and AC groups (arrowheads in Supplemental Fig. S2Aa,b). In contrast, there were X-ray-transparent periodontal spaces between the mesial or distal roots and the alveolar bone surfaces in the PDGF-BB group (Supplemental Fig. S2Ac), as in the NC group (Supplemental Fig. S2Ad). These analyses by reconstructed CT images revealed that numbers of ankylosed replanted teeth tended to be greater in the PBS (4/4) and AC (4/4) groups compared to the PDGF-BB (1/4) group (p < 0.03, χ^2^-test; Supplementary Table S1) 21 days after tooth replantation and compared to the NC (0/4) group at equivalent age (p < 0.005, χ^2^-test; Supplementary Table S1).

Confocal microscopic analysis revealed newly mineralized (calcein-labeled) ankylotic areas that directly connected the mesial and distal root surfaces with the alveolar bone surfaces in the replanted teeth in the PBS and AC groups (Supplemental Fig. S2Ba,b), but not in the PDGF-BB and NC groups (Supplemental Fig. S2Bc,d). Physiological mineralization can be seen in the non-replanted teeth as double calcein labels in the alveolar bone surfaces, cellular cementum, and dentine of the maxillary first and second molars in the NC group (Supplemental Fig. S2Bd). In contrast, disordered mineralization in the replanted teeth was observed in the AC, PDGF-BB, and PBS groups as single labels in the alveolar crest, cementum, and dentine of the mesial or distal roots of the replanted first molars (Supplemental Fig. S2Ba–c).

**Figure 1:**
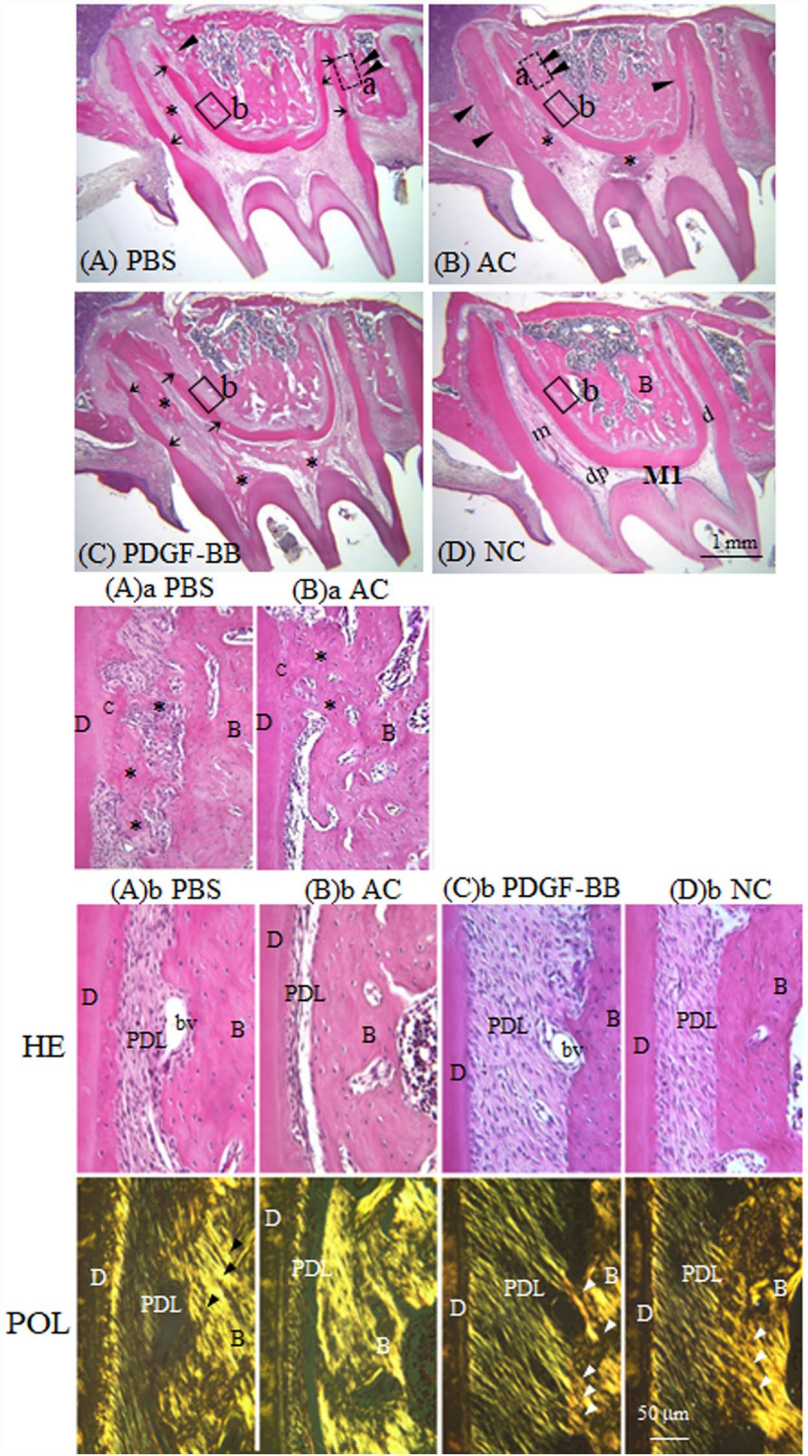
(**A–D**) PDGF-BB effectively suppresses ankylosis and restores well-organized PDL structure after replantation. Haematoxylin and eosin staining of sagittal sections of replanted rat maxillary first molars in the four groups. (**A**) PBS-pretreated, (**B**) atelocollagen-pretreated (AC), (**C**) PDGF-BB-pretreated tooth (PDGF-BB) at 21 days. (**D**) A non-replanted normal tooth (NC). Arrowheads and arrows indicate bony attachments of root surfaces and root resorption lacunae, respectively. Black asterisks indicate bone-like structures in the dental pulp. *m* mesial root, *d* distal root. (**(A)a, (B)a**) Magnified views of ankylosed roots (dotted squares (a) in (**A,B**)) in sagittal sections of PBS- (**(A)a**) and AC- (**(B)a**) pretreated teeth 21 days after replantation. Asterisks indicate bone-like ankylotic tissues. (**(A)b–(D)b**) Magnified views of the periodontal ligament (solid squares (b) in (**A–D**)) in sagittal sections of PBS- (**(A)b**), AC- (**(B)b**) and PDGF- (**(C)b**) pretreated teeth 21 days after replantation, and a normal control tooth (**(D)b**). HE-stained images (HE, upper images) and images under polarized light (POL, lower images). Arrowheads indicate insertions of birefringent collagen fibre bundles into alveolar bones. *B* alveolar bone, *C* cementum, *D* dentine, *bv* blood vessel, *PDL* periodontal ligament.

Next, we investigated the ankylotic tissues histologically. At 21 days after tooth replantation, ankylotic bone-like fusions (marked by arrowheads in Fig. 1A,B and asterisks in Fig. 1Aa,Ba) on the root cementum surfaces were frequently seen in the PBS (Fig. 1A,Aa) and AC (Fig. 1B,Ba) groups. Root resorption lacunae (marked by arrows) were also observed adjacent to the ankylotic regions in the PBS and AC groups (Fig. 1A,B). The PDGF-BB group had fewer ankylotic areas and resorption lacunae on the root surfaces (Fig. 1C). As reported in our previous study^3^, abnormal bone-like structures (marked by asterisks) were observed within the dental pulp in most of the replanted teeth in the PBS, AC, and PDGF-BB groups (Fig. 1A–C), but not in the NC group (Fig. 1D).

### PDGF-BB induced well-organized PDL structure after replantation

Here, we present typical magnified images (boxed areas in Fig. 1A–C) of regenerated soft connective PDL tissues (Fig. 1Ab–Db) on the distal side of the mesial root. In the PBS-treated group at 21 days after replantation, connective tissues were filled in the PDL space (Fig. 1Ab, HE), but birefringent collagen fiber bundles were thin and almost parallel to the root long axis (Fig. 1Ab, POL). In the atelocollagen-pretreated teeth after 21 days, connective tissues were sparse (Fig. 1Bb, HE), and short birefringent collagen fiber bundles were only seen on the cementum surface (Fig. 1Bb, POL). In the PDGF-BB group after 21 days, the PDL connective tissues were well reorganized and the width of the PDL was noticeably greater (Fig. 1Cb, HE) than those in the NC, PBS, and AC groups. Thick birefringent collagen fiber bundles were obliquely or functionally arranged between the root cementum and bone, and their ends were inserted into the alveolar bone and cementum (arrowheads in Fig. 1Cb, POL), as in the normal control PDL (Fig. 1Db, POL).

### PDGF-BB effectively restored the mechanical properties of PDL after replantation

#### Root/bone fractures during biomechanical testing

Next, we performed biomechanical testing of the replanted teeth. To measure the mechanical properties of injured PDL, a replanted tooth was extracted from its socket, and a load-deformation curve of the healing PDL was recorded. During mechanical testing, fractures of tooth roots and/or alveolar bones occur if a higher loading strength is required owing to the ankylotic changes in the PDL. In the NC group, the mesial root of one tooth on days 0 and 14 was fractured during mechanical testing to extract the maxillary first molars from their sockets (Table 1). In the PBS and AC groups on 14 and 21 days, the numbers of root fractures during mechanical testing were significantly greater than those in the PDGF group (p < 0.001, χ^2^-test). In the PDGF-BB group, root fractures occurred in 1/9 and 3/9 replanted teeth at 14 and 21 days, respectively (Table 1). Those numbers of fractures showed no significant differences from that in the NC group (p > 0.1, χ^2^-test). Thus, we successfully obtained a healed PDL without ankylosis from the PDGF-BB group.

**Table 1:** Numbers of fractures of tooth roots and/or alveolar bones during mechanical testing of extracting the replanted or non-replanted teeth from their own sockets.

Days after replantation	Groups			
	NC (40)	PBS (27)	AC (28)	PDGF-BB (30*)
0	1 (10)	–	–	–
7	0 (10)	0 (9)	0 (9)	0 (8)
14	1 (10)	3 (9)	7 (9)^a,b^	1 (9)
21	0 (10)	8 (9)^a^	10 (10)^a^	3 (10)

Number of rats is shown in parentheses.

^a^Significant difference from PDGF-BB group, p < 0.001 (chi-squared test).

^b^Significant difference from PBS group, p < 0.01 (chi-squared test).

*Tooth replantation was unsuccessful in two rats of the PDGF group. In addition, tooth crown of a replanted tooth was fractured due to unsuccessful clamping before mechanical testing in one rat of the PDGF-BB group at 7 days.

#### Load-deformation curves

**Figure 2:**
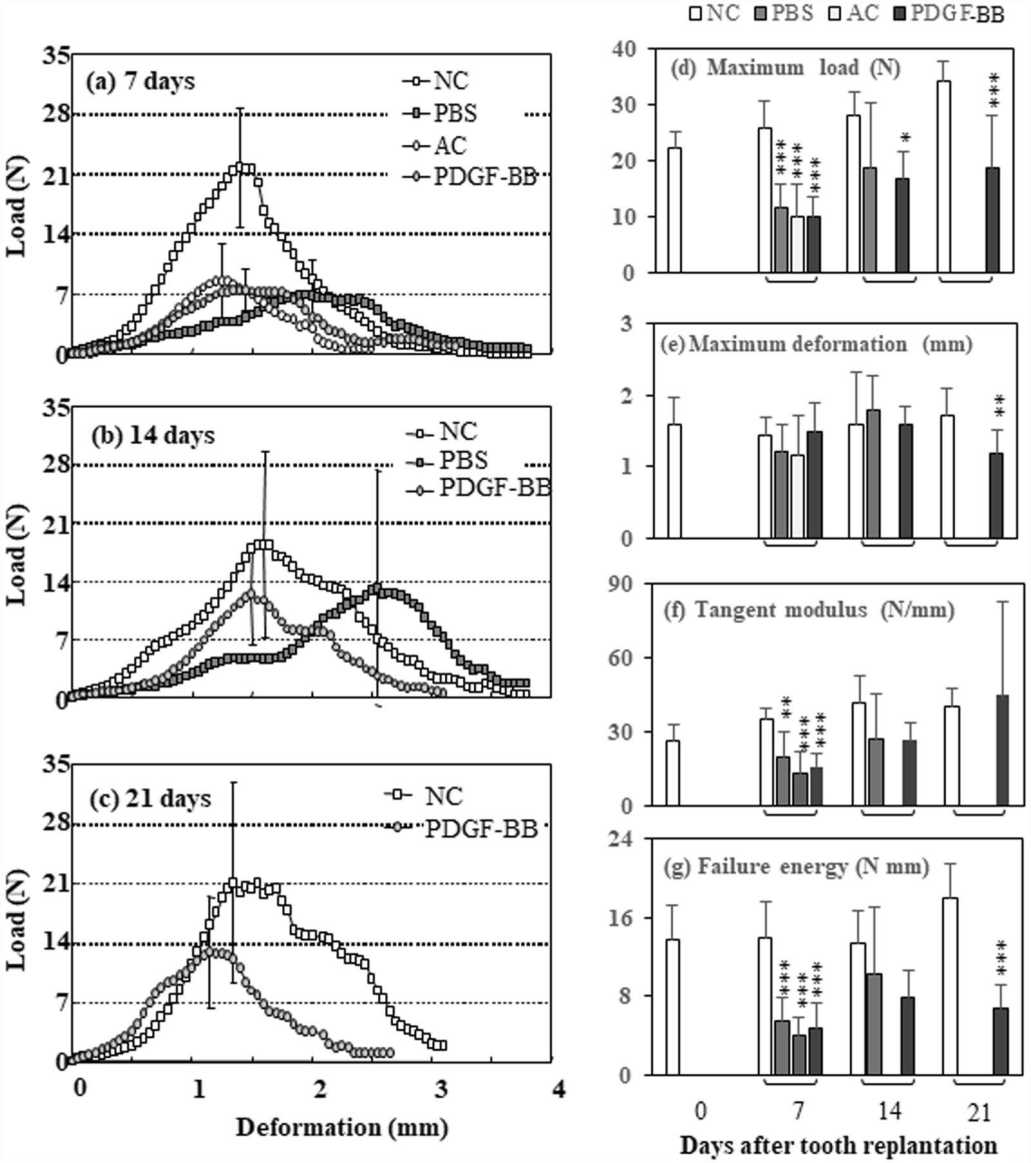
PDGF-BB effectively restores the mechanical properties of PDL after replantation. (**a–c**) Mean load-deformation curves for the healing periodontal ligament of the maxillary first molars at 7, 14 and 21 days after tooth replantation in the PBS, AC, and PDGF-BB groups. The load-deformation curves were obtained by extracting the replanted teeth from their sockets. Data from the NC (non-replanted teeth) group are also presented. Each point represents the mean of 6–10 rats. Vertical bars at the averaged peak value represent ± 1 SD. (**d–g**) Changes in the maximum load (**d**), maximum deformation (**e**), tangent modulus (**f**), and failure energy (**g**) for the healing PDL of the maxillary first molars at 7, 14, and 21 days after tooth replantation in the PBS, AC, and PDGF-BB groups. Data from the NC group are also presented. Each column and bar represents the mean and 1 SD of 6–10 rats. Significant differences from the respective NC group, *p < 0.05, **p < 0.01, ***p < 0.001 (Scheffé method).

Figure 2a–c show the mean load-deformation curves obtained from mechanical testing for the normal PDL in the NC group and for the healing PDL in the PBS, AC, and PDGF-BB groups. At 7 days after tooth replantation, the load levels of the rising parts of the load-deformation curves from the PBS, AC, and PDGF-BB groups were noticeably lower than those from the NC group. From 14 to 21 days, the rising curve of the load levels for the healing PDL from the PDGF-BB group increased and approached those of the normal PDL, but the peak values were lower in the PDGF group than in the NC group.

#### Biomechanical measures

Figure 2d–g shows changes in the maximum load, maximum deformation, tangent modulus, and failure energy for the healing PDL estimated from the load-deformation curves, respectively.

The maximum load indicates the failure load of PDL tissue of a replanted or non-replanted maxillary first molar. The values in the NC group increased significantly during the experimental period (p < 0.01, ANOVA). In the PBS and AC groups, the mean values were considerably lower than those of the NC group at 7 and 14 days (p < 0.001). In the PDGF-BB group, the mean values increased continuously from 7 to 21 days by 90% (ANOVA, p < 0.001). The mean failure load of the PDL in the PDGF-BB group at 21 days recovered to 54% of that of the control.

The maximum deformation indicates the extensibility of PDL tissue. The values in the NC group did not change significantly during the experimental period. The mean values in the PDGF-BB group, as well as in the PBS and AC groups, were similar to those of the NC groups at 7 and 14 days. The mean extensibility of the PDGF-BB group at 21 days recovered to 68% of that of the NC group.

The tangent modulus indicates the stiffness of PDL tissue. Their values in the NC group increased gradually from day 0 to day 14 (ANOVA, p < 0.01) and remained unchanged from day 14 to 21. In the PBS and AC groups, the mean values at 7 (p < 0.001) and 14 days were less than those of the NC group. In the PDGF-BB group, the mean values gradually increased from day 7 to 21. The mean stiffness of the PDL in the PDGF-BB group at day 21 recovered almost to the level of the NC group.

The failure energy indicates the toughness of PDL tissue. Their values in the NC group remained unchanged from days 0 to 14 and increased from days 14 to 21. In the PBS and AC groups, the mean values were lower than that of the NC group. In the PDGF-BB group, the mean value gradually increased from days 7 to 14 by 40%. The mean toughness of the PDL in the PDGF-BB group on day 21 recovered to 44% of that of the NC group.

Soft X-ray analysis of the extracted teeth revealed that tooth replantation completely inhibited root growth, although the non-replanted teeth in the NC group exhibited root growth by 25% during the experimental period (Supplemental Table S2). This observation was confirmed by double calcein labeling on the additive cellular cementum of the two roots of the non-replanted teeth, but not in the replanted teeth (Supplemental Fig. S2B).

Since root growth was observed in the non-replanted teeth but not in the replanted teeth as described above, the root surface area (PDL stress area) is considered to be greater in the NC group than in the PDGF-BB group. Thus, for simplicity, we attempted to standardize the mean maximum load for the root surface area (Supplemental Table S3, Fig. S4). The mean mechanical strength (2.45 N/mm^2^) in the PDGF-BB group was restored to approximately 76% of the control value (3.39 N/mm^2^) at 21 days after tooth replantation.

### PDGF-BB increased PDGF-Rβ positive cells in PDL tissues

**Figure 3:**
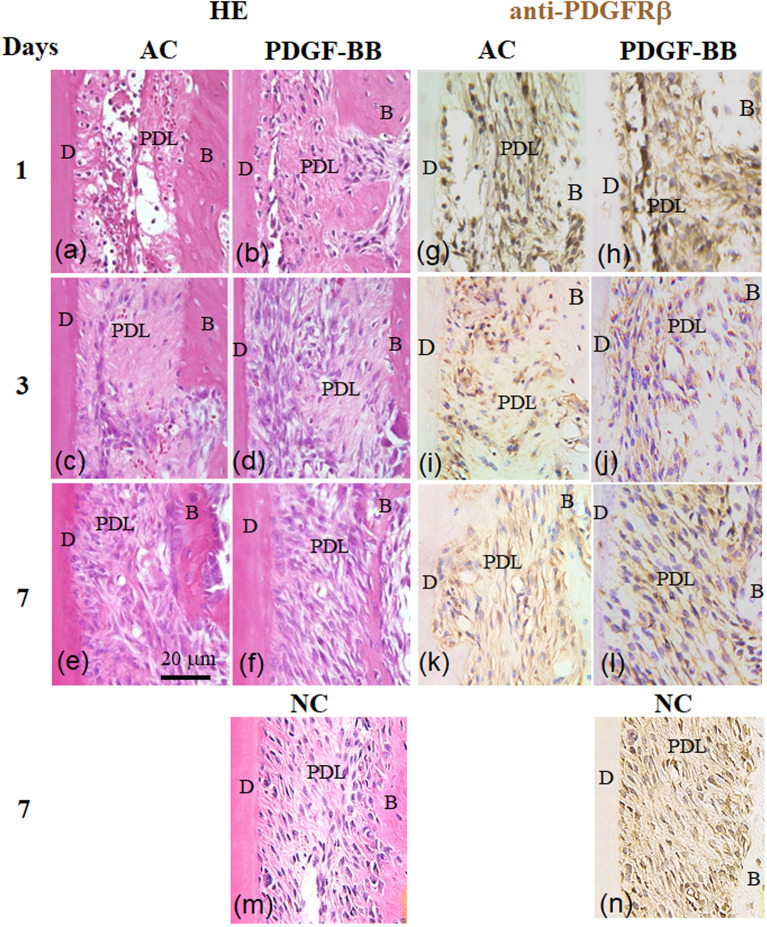
PDGF-BB increases PDGF-Rβ positive cells in PDL tissues. Magnified views of the repairing periodontal ligament (PDL) showing typical repair process from day 1 (**a,b,n,i**), day 3 (**c,d,j,k**) to day 7 (**e,f,l,m,g,n**) after tooth replantation in the AC, PDGF-BB, and NC groups. Sections were stained with haematoxylin and eosin (**a–f,m**), or immunostained with anti-PDGF-Rβ antibody and treated with secondary peroxidase-conjugated antibody (**g–l,n**). *B* alveolar bone, *D* dentine.

To histologically examine the restorative effect of PDGF-BB on the injured PDL at the initial stage after tooth replantation, we compared HE-stained sections in the AC and PDGF-BB groups. On day 1 after tooth replantation, the PDL cells of both groups exhibited pyknotic. The PDL tissues in the AC group were necrotic to a greater extent (Fig. 3a). In contrast, those in the PDGF-BB group were less necrotic than those in the AC group (Fig. 3b). On day 3, the ruptured area of the PDL of the AC group was filled with fewer fibroblast cells (Fig. 3c), whereas in the PDGF-BB group, it was filled with more fibroblast cells (Fig. 3d). The other areas of the PDL in the two groups were filled with soft connective tissue. On day 7, the fibroblast-like cells of the PDL of the AC group were oriented parallel to the long axis of the tooth (Fig. 3e). In contrast, those of the PDGF-BB group became oriented obliquely, as seen in the NC group (Fig. 3g).

Topical PDGF-BB affects tissues by binding to specific cell surface receptors of the target cells and then activating their cellular signaling pathways^8,14,16^. It is well known that PDGF-Rβ, not PDGF-Rα, is upregulated in regenerated tissues during wound healing^14,21^. Thus, we analyzed the localization of PDGF-Rβ positive target cells around the replanted teeth. The cuboidal cementoblasts, fibroblast-like cells, osteoblast-like cells, and osteocytes were PDGF-Rβ-positive around the replanted teeth in all four study groups (Fig. 3g–l,n).

In the AC group, most of the PDL cells were PDGF-Rβ-positive on days 1 and 3, and oriented almost parallel to the tooth long axis on day 7 (Fig. 3g,i,k). In contrast, in the PDGF-BB group, the fibroblastic cells at the ruptured surfaces of the PDL were densely immuno-stained with anti-PDGF-Rβ antibody and the PDGF-Rβ-positive fibroblast-like cells were distributed through the PDL at day 1. They then migrated to a greater degree at the restored zone of the PDL from day 1 through day 7, and became oriented more obliquely on the PDL on day 7 (Fig. 3h,j,l).

### PDGF-BB maintained cell survival and increased BrdU-positive cells in the PDL

**Figure 4:**
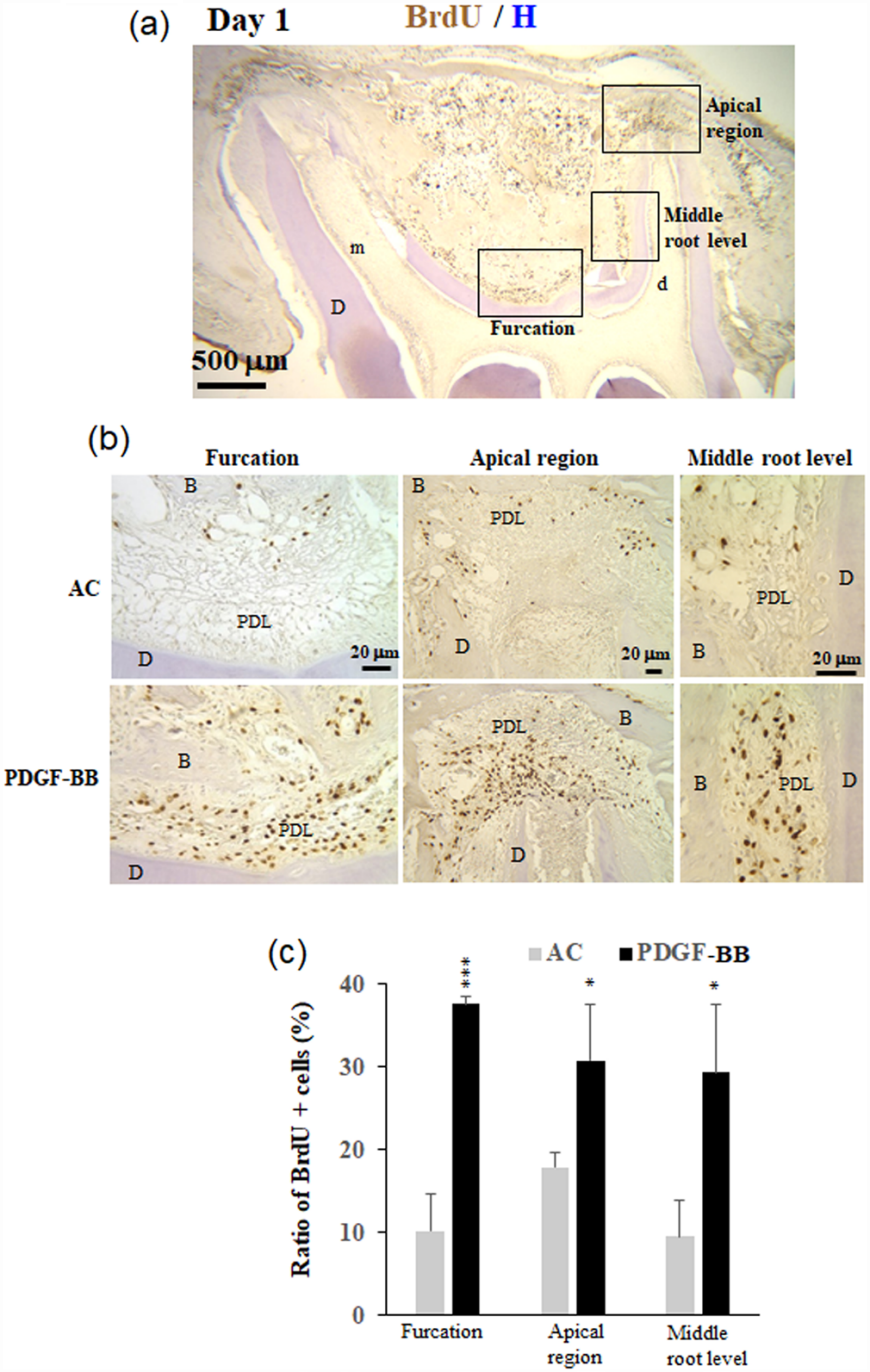
PDGF-BB maintains cell survival and increases the BrdU-positive cells in the PDL. Immunohistochemistry of BrdU-positive cells of sagittal sections of the replanted teeth in the AC, and PDGF-BB groups. The rats were injected with BrdU 1 day after tooth replantation, and the maxillae were isolated 2 h after injection. (**a**) A representative sagittal section showing three regions (squared areas), that is, the periodontal ligaments (PDLs) of furcation, and apical region and middle level of the distal root. (**b**) Magnified views showing the three squared regions in (**a**). (**c**) Ratios of BrdU-positive cells in the PDLs of the three regions (**b**). *m* mesial root, *d* distal root, *B* bone, *D* dentine. Significant differences between the AC and PDGF-BB groups, *p < 0.05, ***p < 0.01 (*t*-test).

PDGF is well known to be present at wound sites and has potent effects on growth, chemotaxis, and matrix production in the healing process^6^. In this study, we examined the effect of PDGF-BB on cell proliferation (Fig. 4). BrdU-positive cells were clearly observed in the periodontal regions of the mesial and distal roots, the bone marrows between the two roots, and the gingival tissues of the replanted teeth on day 1 in the four groups (Fig. 4a). Few BrdU-positive cells were observed on days 3 and 7 in the four groups (data not shown), indicating that cell proliferation occurred markedly at day 1, but not on days 3 and 7 after replantation. Thus, we focused on BrdU-positive cells in the periodontal regions of the apical areas, furcation, and PDL at the middle level of the distal roots on day 1. More BrdU-positive cells in these three regions were observed in the PDGF-BB group than in the AC group (Fig. 4b). The ratios of BrdU-positive cells in these three areas were significantly greater in the PDGF-BB group than in the AC group (Fig. 4c; p < 0.001–0.05).

### PDGF-BB decreased osteocalcin-positive cells around the replanted teeth, and inhibited β-catenin accumulation in the nucleus in vivo

**Figure 5:**
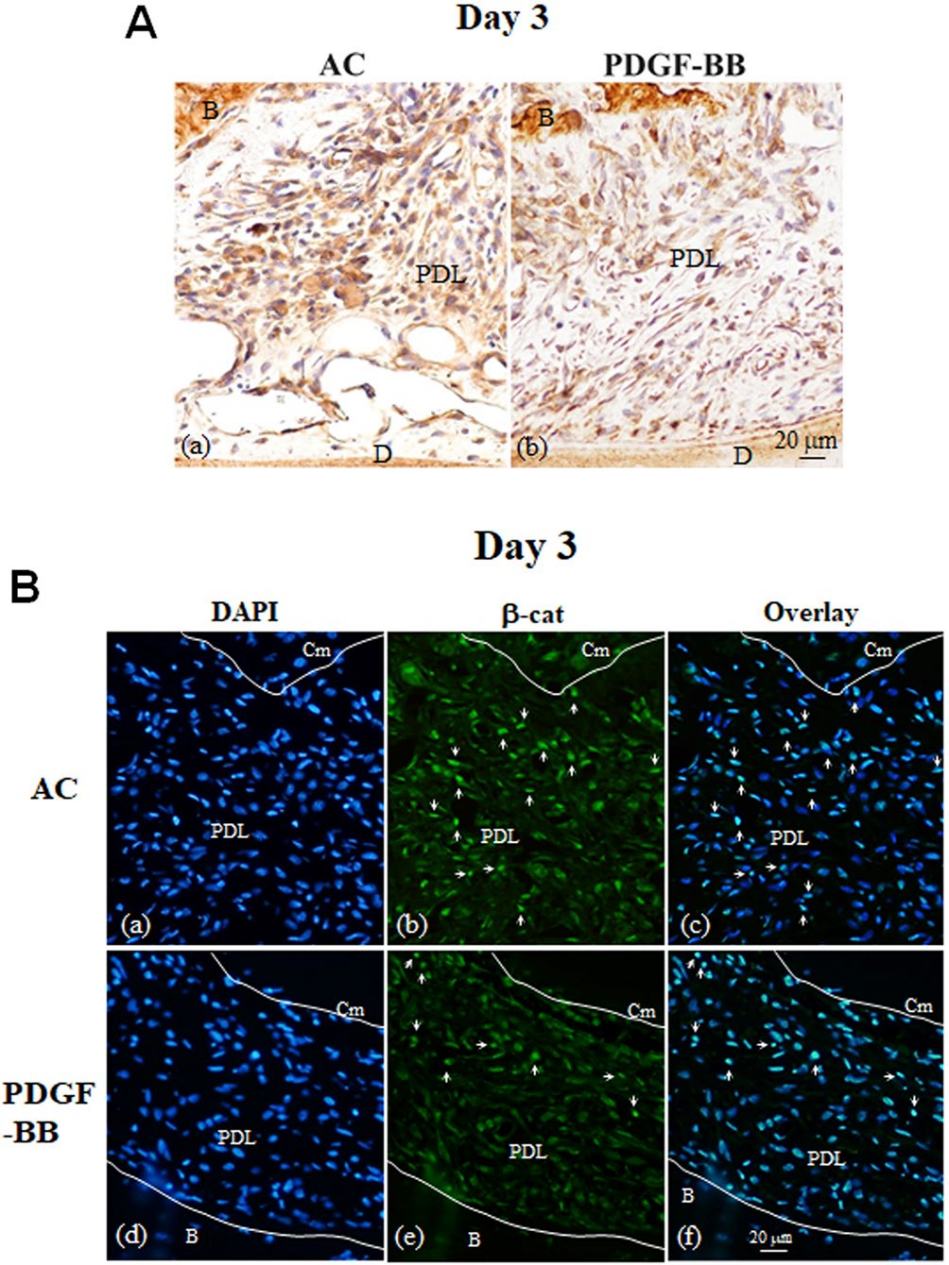
PDGF-BB decreases osteocalcin-positive cells and β-catenin accumulation in nucleus in vivo. (**A**) Representative images of sections immunostained for osteocalcin (OCN). Sections were immunostained with primary anti-OCN antibody and peroxidase-conjugated secondary antibody, and the immune complex was visualized with DAB substrate. The sections were then counterstained with haematoxilin. Pictures were taken of periodontal ligament (PDL) of the mesial root of the replanted teeth in the AC (a) and PDGF-BB (b) groups 3 days after tooth replantation. (**B**) Representative images of sections immunostained for β-catenin. Sections were incubated with primary anti-β-catenin antibody and secondary Alexa 488-conjugated antibody, and then counterstained with DAPI. DAPI (Ba and Bd), ß-catenin (Bb and Be), and overlay (Bc and Bf) images of the periodontal tissues. Pictures were taken of PDL of the mesial root of the replanted teeth day 3 in the AC (a-c) and PDGF-BB (d-f) groups. White arrows indicate nuclear b-catenin positive cells. *B* bone, *Cm* cementum, *D* dentine.

To observe how ankylotic tissues are formed after tooth replantation, tissue sections were immunostained with osteocalcin antibody, which detects osteoblastic differentiation. We found more osteocalcin-positive cells in the periodontal tissues in the AC group, but few in the PDGF-BB group on day 3 after tooth replantation (Fig. 5Aa–Ab).

As it has recently been reported that sustained canonical Wnt/β-catenin signaling in periodontal tissues causes dental ankylosis^20^, we immunostained sections with the anti-β-catenin antibody^20^. Both the AC and PDGF-BB groups had cytoplasmic β-catenin positive cells in the periodontal ligament tissues at days 3 (Fig. 5B) and 7 (Supplementary Fig. S5) after tooth replantation. By immunofluorescence analyses, more nuclear accumulation of β-catenin was observed in the periodontal cells near the tooth surfaces in the AC group (Fig. 5Ba–c). However, this was observed less in the PDGF-BB group (Fig. 5Bd–f).

### PDGF-BB increased cell proliferation and migration in vitro

**Figure 6:**
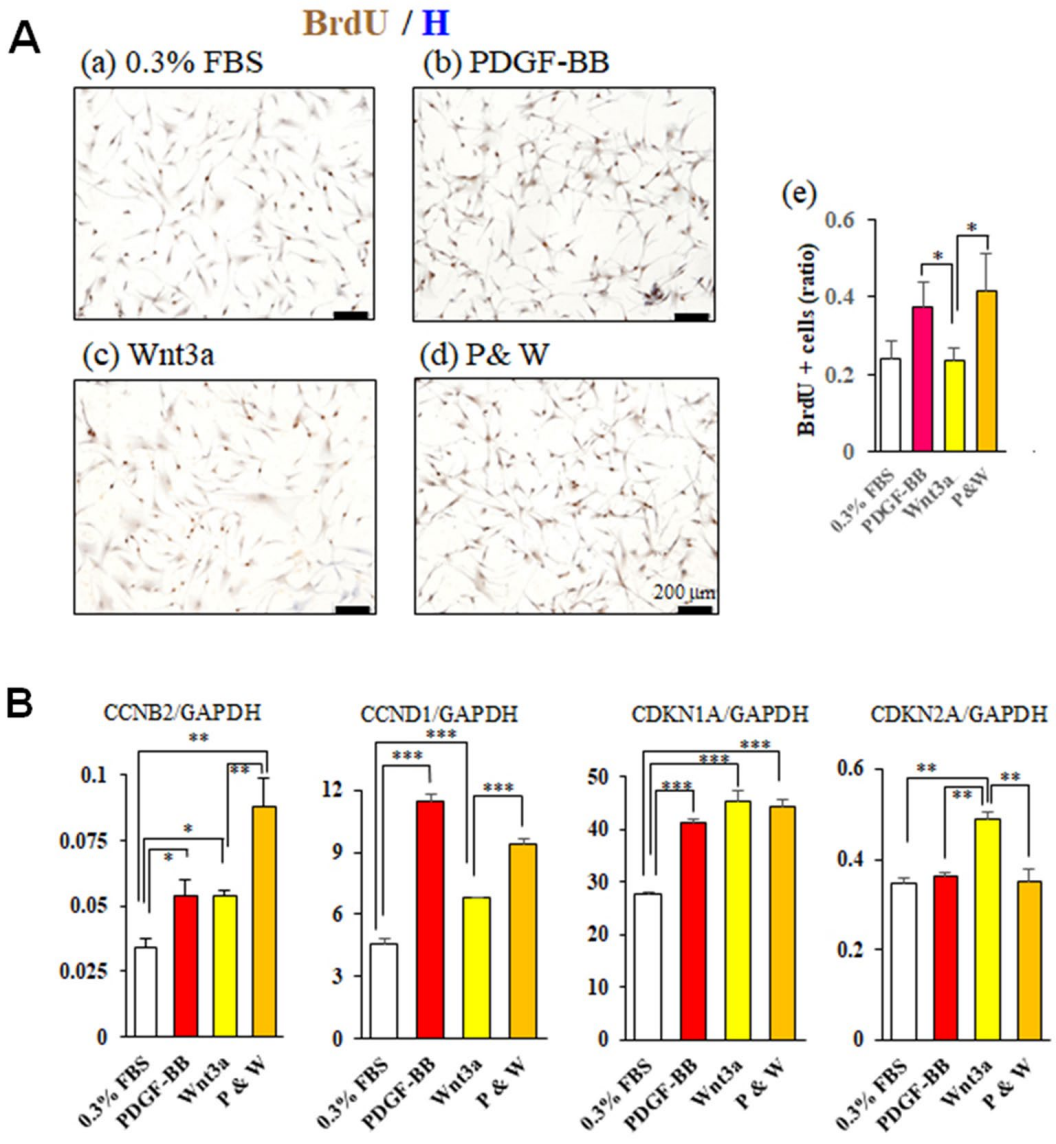
PDGF-BB increases cell proliferation in vitro. (**A**) Effect of PDGF-BB on BrdU incorporation. DMEM contained with 0.3% FBS and BrdU. Subconfluent human PDL fibroblasts were cultured under DMEM only (0.3% FBS, (a)), PDGF-BB (5 ng/mL, (b)), Wnt3a (25 ng/mL, (c)), or their combination (P&W, (d)) for 24 h. Scales, 200 µm. (e) BrdU positive cell ratio. Mean ± 1 SD are shown. Significant differences between two groups, *p < 0.05 (Fisher PLSD method). (**B**) Effect of PDGF-BB on gene expressions of CCNB2, CCND1, CDKN1A, and CDKN2A. Mean ± 1 SD are shown. Significant differences between two groups, *p < 0.05, **p < 0.01, ***p < 0.01 (Fisher PLSD method).

To examine the molecular mechanisms underlying the effects of PDGF-BB on PDL, we used human periodontal fibroblasts and analyzed the effects on cell migration, proliferation, and differentiation in vitro. Since an interaction between canonical Wnt signaling and PDGF-BB is implicated in in vivo results (Fig. 5), we analyzed the effect of Wnt on human PDL fibroblasts. Figure 6A shows representative BrdU-immunostained microscopic images from human PDL fibroblasts cultured in 0.3% FBS (a), 5 ng/mL PDGF-BB (b), 25 ng/mL Wnt3a (c), and PDGF-BB & Wnt3a (P&W, d). The BrdU-positive cell ratios in the PDGF-BB and PDGF-BB&Wnt3a groups are greater than those in the 0.3% FBS group and the Wnt3a group (p < 0.05; Fig. 6Ae). In addition, the expression levels of cyclin genes CCNB2 and CCND1 were increased in the PDGF-BB, Wnt3a, and PDGF-BB&Wnt3a groups compared to the control (p < 0.05–0.01; Fig. 6B) and both expression levels were higher in the PDGF-BB&Wnt3a group. The gene expression of cyclin inhibitor CDKN2A was upregulated in the Wnt3a group compared to the control (p < 0.01), which was suppressed in the PDGF-BB&Wnt3a group. CDKN1A gene expression levels was upregulated in the PDGF-BB, Wnt3a, and PDGF-BB&Wnt3a groups (p < 0.001) while no differences among the three groups were found.

**Figure 7:**
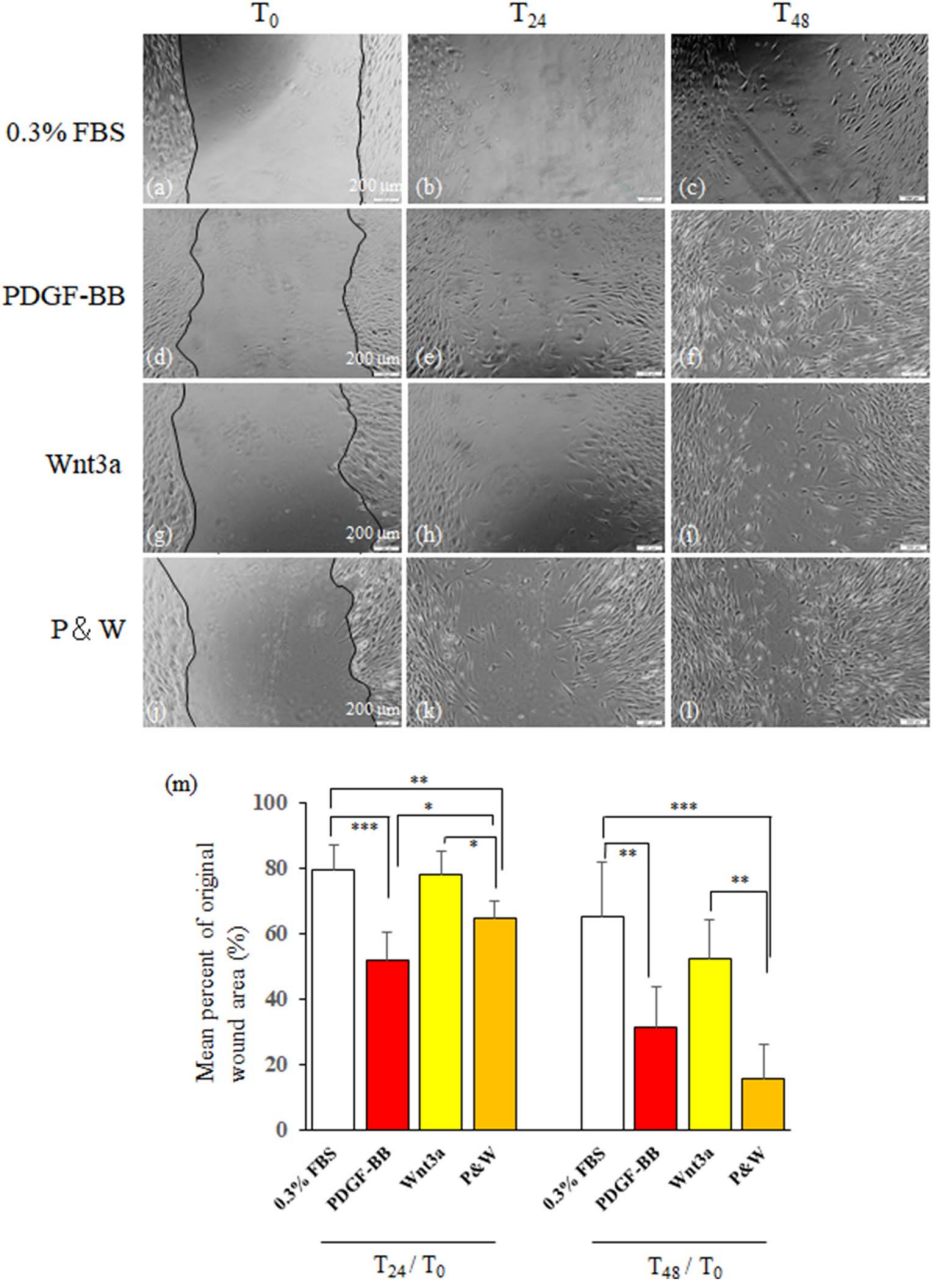
PDGF-BB increases cell migration in vitro. Effect of PDGF-BB on wound scratch assay. Confluent human PDL fibroblasts were subjected to injury by scratching with a plastic pipette tip. The cells were then cultured under 0.3% FBS (solvent, (**a–c**)), PDGF-BB (5 ng/mL, (**d–f**)), Wnt3a (25 ng/mL, (**g–i**)), or their combination (P&W, (**j–l**)). Pictures were taken at 0 h (T_0_; (**a,d,j,g**)), 24 h (T_24_; (**b,e,h,k**)), and 48 h (T_48_; (**c,f,i,l**)). (**e**) Ratios (T_24_/T_0_) and (T_48_/T_0_) of wound area at T_24_ and that at T_48_ to original wound area at T_0_. Mean ± 1 SD are shown. Significant differences between two groups, *p < 0.05, **p < 0.01, ***p < 0.01 (Fisher PLSD method).

Next, we examined the effects on cell migration. Figure 7 shows representative phase-contrast microscopic views of wound scratches of human PDL fibroblasts cultured in 0.3% FBS (a–c), 5 ng/mL PDGF-BB (d–f), 25 ng/mL Wnt3a (g–i), and PDGF-BB&Wnt3a (P&W, j–l) at 0 (T_0_), 24 h (T_24_), and 48 h (T_48_) after the formation of wound scratch. More fibroblasts in the wound areas were seen in the PDGF-BB, and PDGF-BB and Wnt3a groups than in the 0.3% FBS and Wnt3a groups, indicating that PDGF-BB accelerates the migration of human PDL fibroblasts into wound scratches. Figure 7m shows mean ratio of wound area/original wound area at T_24_ and T_48_ in each group. T_24_/T_0_ in the PDGF-BB, and PDGF-BB and Wnt3a groups are lower than those in the 0.3% FBS and Wnt3a groups (p < 0.05–0.001). T_48_/T_0_ values decreased compared to T_24_/T_0_ in each group and those in the PDGF-BB, and PDGF-BB and Wnt3a groups are lower than those in the 0.3% FBS and Wnt3a groups (p < 0.01–0.001).

### PDGF-BB attenuated effects of Wnt signaling on differentiation of PDL fibroblasts in vitro

**Figure 8:**
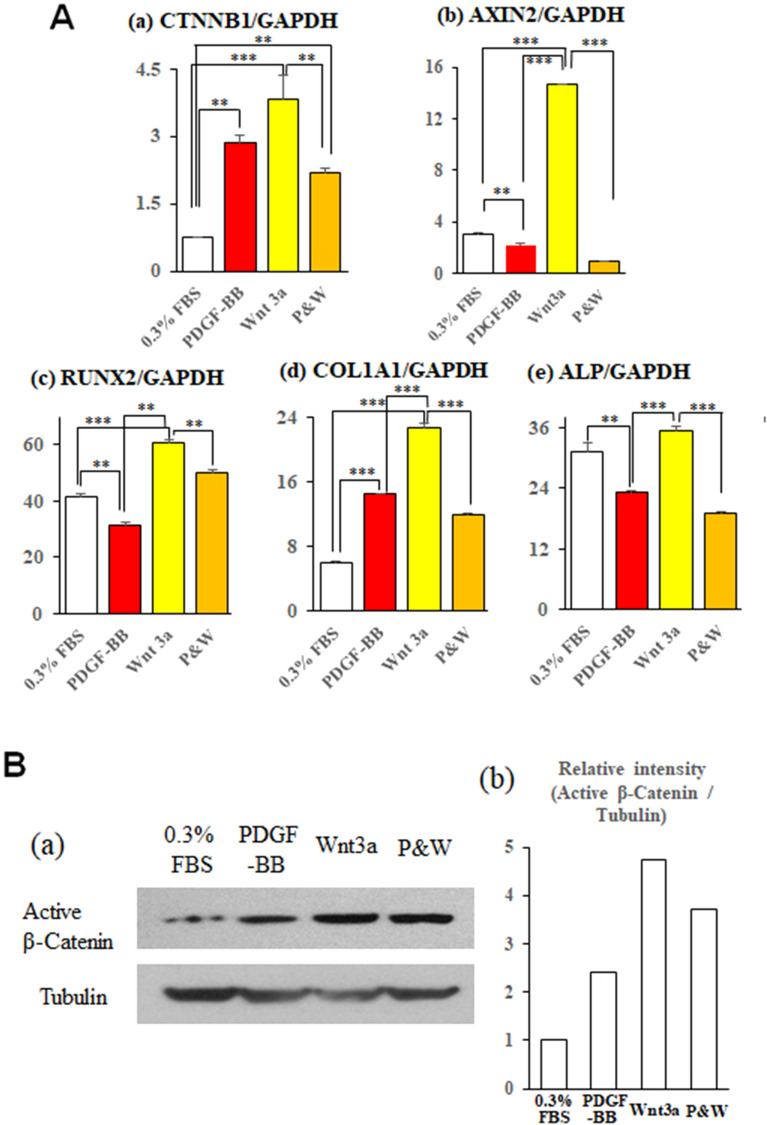
PDGF-BB attenuates effects of canonical Wnt signaling on PDL fibroblasts in vitro. (**A**) Gene expressions of CTNNB1 (a), AXIN2 (b), RUNX2 (c), COL1A1 (d), and ALP (e) in human PDL fibroblasts treated with 0.3% FBS, PDGF-BB (5 ng/mL), Wnt3a (25 ng/mL), or their combination (P&W) for 4 days. Significant differences between two groups, *p < 0.05, **p < 0.01, ***p < 0.001 (Fisher PLSD method). (**B**) (a) Protein level of active form of β-catenin were measured by western blotting. Lysate was generated from human PDL cells at 24 h after each treatment. Tubulin was used as a loading control. Original blots are presented in Supplementary Fig. S6. (b) The densities of the active β-catenin protein bands were normalized relative to Tubulin protein bands.

PDGF-BB decreased the gene expression of RUNX2 (p < 0.01) and ALP (p < 0.01), but increased the expression of CTNNB1 (p < 0.01) and COL1A1 (p < 0.001) (Fig. 8Aa–d). Wnt3a upregulated the expression of CTNNB1 (p < 0.001), AXIN2 (p < 0.001), RUNX2 (p < 0.001), and COL1A1 (p < 0.001). The addition of PDGF-BB to Wnt3a downregulated the expression of CTNNB1 (p < 0.01), AXIN2 (p < 0.001), RUNX2 (p < 0.01), COL1A1 (p < 0.001), and ALP (p < 0.01). Western blot revealed that active (dephosphorylated) β-catenin protein was increased in the PDL fibroblasts treated with Wnt3a compared to those treated with PDGF-BB (Fig. 8B). The addition of PDGF-BB to Wnt3a decreased active β-catenin protein.

## Discussion

### Ankylosis in replanted teeth

When large areas of the PDL are traumatized in tooth replantation, competitive wound healing processes begin between mesenchymal stem cells destined to form bone and those capable of forming PDL fibers and the cementum, resulting in either transient or permanent ankylosis^1,2^. PDL also contains cells with osteoblastic properties expressing mineralization markers (ALP, BSPII, etc.) that form calcium nodules and are influenced by osteoinductive factors such as BMPs, IGF-1, and TGF-β1^22^.

The histologic, CT, and calcein labeling observations demonstrated that pretreatment of tooth roots with PDGF-BB effectively reduced the occurrence of ankylosis after replantation of rat teeth (Fig. 1, Table 1, Supplemental Fig. S2, Table S1). One of the mechanisms underlying the inhibitory effect of PDGF-BB on ankylosis could be the suppression of osteoblastic differentiation from PDL cells; PDGF-BB suppressed osteocalcin-positive cells in vivo (Fig. 5A), and Wnt3a induced bone marker expressions in PDL cells in vitro (Fig. 8A). It has been reported that continuous exposure to PDGF-BB accelerated cell proliferation in rat calvaria but stimulated neither the rate of collagen synthesis nor bone matrix formation^23^. Furthermore, continuous treatment with PDGF-BB caused dose-dependent decreases in ALP activity and mineralized bone matrix in osteoblast culture^24^ and in human PDL cell culture^25^. Depletion of PDGF-R β in bone marrow mesenchymal stromal cells decreased mitogenic and migratory responses, and enhanced osteogenic differentiation, whereas PDGF-BB, but not PDGF-AA, inhibited osteogenic differentiation in the same cells with PDGF-R β^26^. Therefore, it is suggested that migration of mesenchymal cells from the periodontal tissues and their surrounding alveolar bones to the injured PDL tissues may occur, but the osteogenic differentiation of bone marrow-derived and PDL mesenchymal cells may be inhibited in the PDGF-BB group (Figs. 5, 8).

Recently, Wu et al. reported^20^ that aberrantly elevated canonical Wnt signaling in dominant active (da) β-cat^Ot^ mice^27^ is responsible for dental ankylosis. These authors confirmed that alveolar bone osteocytes and cementocytes express a stabilized form of β-catenin in da β-cat^Ot^ mice. We hypothesized that PDGF-BB reduces the formation of ankylotic tissues in the injured PDL of replanted teeth by inhibiting the canonical Wnt signaling pathway. Thus, we first performed the same staining method for β-catenin as in Wu’s study (Supplemental Fig. S5). Further immunofluorescence revealed that more β-catenin signals were localized to the cell nucleus in some of the cementocytes and PDL cells near the tooth and bone surfaces of the replanted teeth in the AC group, but less in the PDGF-BB group. In vitro analyses showed that treatment of human PDL fibroblasts with Wnt3a increased down-stream gene expressions of canonical Wnt signaling and osteogenic gene expressions, and the addition of PDGF-BB decreased their expressions (Fig. 8A). Western blot analysis indicated that the addition of PDGF-BB inhibited the Wnt3a-induced increase of active dephosphorylated β-catenin protein in human PDL cells (Fig. 8B). Thus, we suggest that PDGF-BB inhibits the canonical Wnt signaling in injured periodontal tissues, which is one of the mechanisms for prevention of tooth-bone ankylosis after tooth replantation (Supplemental Fig. S7).

### Better restoration of the injured PDL by topical PDGF-BB

It has previously been shown that PDGF-R β signaling, not PDGF-Rα signaling, distinctively induces proliferative and migratory responses of PDL cells^14,21,22,28^. Immunohistochemical analysis revealed that more target PDGF-R β cells existed in the PDL tissues and that cell proliferation was promoted in the PDL tissues of the PDGF-BB group more than in those of the AC group after tooth replantation.

The cyclin D complex phosphorylates the target protein, progressing the cell cycle from the G1 to the S phase; the cyclin B complex was reported to regulate the G2/M phase transition^29^. CDKN1A suppresses cyclin A and CDK partner activities in G1, and CDKN2A suppresses cyclin D and CDK partner activity in G1^30^. In the present study, we found that expression levels of cyclins D and B genes in the PDGF-BB group were upregulated whereas in the PDGF-BB group cyclin inhibitor CDKN2A did not change and upregulated level of CDKN1A gene expression was similar to that in the Wnt3a group (Fig. 6B). Therefore, PDGF-BB may promote cell growth through regulating the cell cycle^31,32^ and the DNA synthesis (Fig. 6A)^29^. Our wound scratch assay further showed that PDGF-BB effectively promoted the migration of human PDL fibroblasts to in vitro wounds. It can be argued that these responses via PDGF-BB/PDGF-Rβ signaling of the injured PDL are a prerequisite for the next step in the repair process of the injured PDL.

From a functional point of view, the next important steps in wound repair are the synthesis and functional organization of extracellular matrix proteins, such as collagen type I, by connective tissue cells^17,19^. Although we did not measure newly synthesis of collagen type I in the injured PDL after tooth replantation, in vitro analysis confirmed that PDGF-BB upregulated mRNA expression of COL1A1 in human PDL cells (Fig. 8Ad). As the ankylosis between the tooth roots of the replanted teeth and their surrounding alveolar bones was effectively prevented in the PDGF-BB group, the replanted teeth could be loaded by physiological occlusive forces, and the repopulated PDL tissues were mechanically stimulated. Thus, the better reorganization and functionally-oriented thick fiber bundles could be recovered in the PDL tissues of the replanted teeth of the PDGF-BB group (Figs. 1, 3), as in previous animal studies^3,4,18^. In contrast, the replanted teeth of the PBS and AC groups with ankylotic regions exhibited thin periodontal ligaments with scant fiber bundles. Our biomechanical measurement (Fig. 2) uniquely adds evidence that topical PDGF-BB effectively recovers the mechanical properties of the healing PDL after tooth replantation (Fig. 2, Supplemental Figs. S4, S7).

In the rat tooth replantation model, mineralized tissues are frequently formed in the dental pulp as reported previously^3,41^. Similarly, pulpal calcification is frequently observed during healing process after replantation of avulsed immature teeth in human^42^. In the present study we also observed abnormal bone-like structures within the dental pulp even in the replanted teeth of the PDGF-BB group, suggesting that pulpal calcification after replantation without endodontic treatment could not be hindered by tooth immersion in the PDGF-BB containing solution. Further studies are needed to explore regulatory mechanisms of pulpal calcification after replantation from a clinical point of view.

In conclusion, the present analyses indicate that the promoted repair of the injured PDL by topical PDGF-BB is due to the acceleration of cell proliferation and migration via PDGF-Rβ signaling and the prevention of tooth ankylosis through inhibition of canonical Wnt activation, leading to occlusal mechanical loading of the periodontal tissues and subsequent functional restoration of the PDL tissues. This suggests a possible clinical application of PDGF-BB to prevent ankylosis and promote proper regeneration of PDL after tooth replantation.

## Methods

All animal experimental procedures were approved by the Animal Care Committee of Tsurumi University School of Dental Medicine. All experiments using rats were carried out in compliance with relevant guidelines and regulations including the ARRIVE guidelines^33^.

### Tooth replantation

The procedure of tooth replantation was described in detail previously^3^ (Supplemental Fig. S1A). Briefly, under anesthesia the left maxillary first molars of male Wistar rats at 41–43 days of age were extracted, rinsed in phosphate-buffered saline (PBS), placed in either PBS (PBS group), 0.7% atelocollagen (AC; KOKEN, Tokyo, Japan; dissolved in PBS), as a local carrier^5^ of PDGF-BB (AC group), or PDGF-BB (5 μg/0.7% AC 50 μL/tooth; recombinant rat PDGF-BB, R&D Systems, MN, USA) (PDGF-BB group) for 5 min, and replanted into their sockets. When the tooth crown or root was broken during the replantation procedures, the data from such rats were excluded. In normal control (NC) group of rats, no extraction or replantation of their maxillary first molars was performed.

### Morphological analysis of healing PDL

Tooth replantation was performed as described above. At 21 days after replantation, the PBS (n = 4), AC (n = 4), and PDGF-BB (n = 4) groups of animals were perfused with 4% paraformaldehyde under anesthesia. The NC group (n = 4) was perfused on the same day. The maxillae were dissected and placed in the same fixative at 4 °C.

#### Peripheral quantitative computed tomography

Each maxilla containing a replanted tooth was horizontally scanned at 60 μm intervals with a peripheral quantitative computed tomography (pQCT) system to observe the replanted tooth and its surrounding bone^3^. Two- and three-dimensional analyses were performed to determine ankylosis and resorption of the bones and roots in the replanted and NC teeth using a DICOM viewer (RadiAnt, Medixant, Poznań, Poland).

#### Histology

The maxillae were decalcified in 14% EDTA for 3 weeks, dehydrated, and embedded in paraffin. Mesio-distal longitudinal sections were cut serially with a microtome setting of 6 μm, were stained with haematoxylin and eosin. For polarized light microscopy, sections were examined under crossed polaroids to observe birefringent collagen fibres according to previous studies^34,35^.

### Calcein labeling

Tooth replantation was done as described above. Animals in the PDGF-BB (n = 2), AC (n = 3), PBS (n = 2), and NC (n = 2) groups received s.c. injections of calcein (3 mg/kg b. w.) on day 0 and day 14 after tooth replantation. Undecalcified sections were prepared^36^ from maxillae dissected on day 23, calcein fluorescence labels being observed with a confocal laser scanning microscope.

### Biomechanical testing of healing PDL

Tooth replantation was performed as described previously. Tooth replantation was unsuccessful in two rats in the PDGF group. At 7, 14, and 21 days after tooth replantation, the PBS (n = 27), AC, (n = 28) and PDGF-BB (n = 30) groups of animals were killed at 7, 14, and 21 days after replantation. In the NC group (n = 40), rats were killed at 0, 7, 14, and 21 days. In each rat, immediately after death, the maxilla was dissected out, the adherent soft tissues were removed, and the maxilla was kept in saline at 4 °C until mechanical testing. A load-deformation curve of the healing PDL was recorded by extracting a replanted tooth from its socket at a speed of 5 mm/min (Supplemental Fig. S1C) using a material testing machine^37^. All experiments were performed at room temperature (22–26 °C). The time between killing the animals and recording the load-deformation curves ranged from 15 to 49 min. Mechanical testing failed in one specimen of the PDGF group at 7 days due to unsuccessful clamping of the tooth crown.

Radiographs of the extracted teeth and maxillae were obtained using a soft X-ray apparatus (Type EMB, Softex, Tokyo, Japan). Fractures of alveolar bones and/or teeth during mechanical testing and broken periodontal ligament adhering to root surfaces of extracted teeth were examined on radiographic images and by stereomicroscopic observation of the extracted teeth and alveolar bones.

From each load-deformation curve, the following biomechanical measures were estimated^38^: (1) maximum load (the peak value; failure load); (2) maximum deformation (the deformation at the maximum load; extensibility), (3) tangent modulus (the slope of the linear region of the curve; stiffness), and (4) failure energy (the area between the load-deformation curve and the x-axis to the peak value; toughness).

### Histochemistry of healing PDL

Thirty-four rats were used. Tooth replantation was done as described previously. They were divided into NC group, PBS group, AC group, and PDGF-BB group. Each group was further divided into day 1 subgroup, day 3 subgroup, and day 7 subgroup. In the day 1 subgroup, day 3 subgroup, and day 7 subgroup, each rat was injected with BrdU (Sigma, MO, USA; 30 mg/kg b.w., ip) 1 day, 3 days, and 7 days after tooth replantation, respectively. Each rat was perfused with 4% paraformaldehyde at 2 h after the BrdU injection, and then its maxilla was dissected. The maxillae were decalcified in 14% EDTA, and then embedded in paraffin. Sagittal sections were serially prepared, and then stored at − 20 °C until staining. Some sections were stained with haematoxylin and eosin.

#### Immunohistochemistry for BrdU

The staining procedure was described in detail previously^36^. Numbers of BrdU-positive cells and of nuclei were counted using Image J in the PDL tissues of furcation, apical and middle levels of the distal root of the replanted teeth. Then, ratio (%) of the BrdU-positive cells was calculated as (number of the BrdU-positive cells/number of cells with haematoxylin-stained nuclei) × 100.

#### Immunohistochemistry for PDGF-Rβ

Deparaffinized sections were incubated with anti-PDGF-Rβ primary antibody (rabbit polyclonal, SC339, Santa cruz, Calfornia, USA) and then incubated with peroxidase-conjugated secondary antibody (simple stain rat MAX-PO, Nichirei, Tokyo, Japan). DAB substrate solution was added to produce brown precipitate. The sections were counterstained with haematoxylin. Localization and orientation of the PDGF-Rβ-positive cells were analyzed in the AC and PDGF-BB groups.

#### Immunohistochemistry for osteocalcin

The staining procedure was described in detail previously^36^. Briefly, deparaffinized sections from the AC and PDGF-BB groups were incubated with a primary antibody against bovine osteocalcin (OC4-30, Takara, Ohtsu, Japan) and incubated with peroxidase-conjugated secondary antibody. The immune complex was visualized with DAB substrate, and the sections were counterstained with haematoxylin.

#### Immunohistochemistry for β-catenin

Deparaffinized sections from the days 3 and 7 subgroups of the AC and PDGF-BB groups were incubated with a primary antibody against mouse β-catenin (610154, BD biosciences, Tokyo, Japan) and incubated with Alexa 488-conjugated secondary antibody. The sections were counterstained with DAPI. Alternatively, after incubation with the primary antibody sections were incubated with peroxidase-conjugated secondary antibody and then immune conplex was visualized with DAB substrate, and the sections were counterstained with haematoxylin.

### Cell culture

Human PDL fibroblasts were purchased from Lonza (CC07049 HPdLF, Lonza, Tokyo, Japan). The cells were cultured under DMEM containing 10% FBA and 100 mg/mL penicillin and 100 IU/mL streptomycin (P/S). After the cells reached subconfluence, the medium was replaced with DMEM containing 0.3% FBS and P/S. The next day, PDGF-BB (5 ng/mL dissolved in DMEM with 0.3% FBS; recombinant human PDGF-BB, Oriental yeast, Tokyo, Japan), Wnt3a (25 ng/mL dissolved in DMEM with 0.3% FBS; recombinant mouse Wnt3a, R & D, Minneapolis, USA), or their combination was added to the medium. Each concentration was determined in preliminary experiments (0.1–10 ng/mL for PDGF-BB; 25–100 ng/mL for Wnt3a). The medium containing each factor was replaced every 2nd day.

### BrdU incorporation assay

The human PDL fibroblasts were cultured under DMEM with 0.3% FBS containing BrdU and each factor for 24 h. The staining procedure was described in detail previously^36^. Numbers of BrdU-positive cells and of nuclei were counted using Image J.

### Wound scratch assay

Wound scratch assay was performed as described previously^14,39^. After confluent cultured cells grown in 24-well plates were obtained, the medium was replaced with 0.3% FBS containing DMEM. The next day, the cells were scratched with a 1-mL plastic pipette tip and then treated with each factor for 48 h. The cells were viewed with an inverted phase contrast microscope. Wounds at time 0 h (T_0_), 24 h (T_24_), and 48 h (T_48_) after addition of each factor were photographed, and wound area was measured at each time point by using Image J software (version 1.53 k). Then, ratio (T_24_/T_0_ or T_48_/T_0_) was calculated by dividing wound area at T_24_ or T_48_ with its original wound area at T_0_.

### Reverse transcription real-time PCR

The cells were plated in 6-well plates and treated as described for the wound scratch assay. On day 4, total RNA was extracted and real-time RT-PCR analysis was performed^40^. The primer sequences are listed in Supplemental Table S3.

### Western blot

Western blot analysis was performed as previously described^40^. Briefly, 24 h after the switch to each medium the cells were washed in ice-cold PBS and solubilized in lysis buffer supplemented with the protease and phosphatase inhibitor was dissolved. The lysed proteins were separated by 10% SDS-PAGE pre-cast gels and electrotransferred to PVDF membranes. Membranes were incubated with anti-active β-catenin (an active form of β-catenin lacking phosphorylation at S37/Thr41, clone 8E7, Millipore, Temecula, CA, USA)^43^ or anti-Tubulin (Millipore-Sigma, St. Louis, MO, USA), followed by incubation with horseradish peroxidase-conjugated secondary antibody. Immunoreactive bands were detected by chemiluminescence. The density of the blots was quantified using Image Studio Lite v.5.2 software (LI-COR Biosciences, Lincoln, NE, USA). The densities of the Active β-Catenin protein bands were normalized relative to Tubulin protein bands from the same sample and the relative intensity were calculated relative to control.

### Statistics

The chi-squared (χ^2^) test was used to examine differences in the numbers of ankylosed teeth at 21 days after tooth replantation and in the numbers of fractures during mechanical testing between the two groups. The changes in the mean values of mechanical measures at 7, 14, and 21 days were examined by a one-way analysis of variance (ANOVA) and a post hoc test (Scheffé method) were further used to examined the differences of the mean values between any two groups of the four groups. The mean values of ratios of BrdU positive cells in the AC and PDGF-BB groups were compared by *t*-test. The mean values of BrdU positive cell ratio, wound area, gene expressions of HPDL fibroblasts in the 0.3% FBS, PDGF-BB, Wnt3a, and PDGF-BB&Wnt3a groups were examined by ANOVA and a post hoc test (Fisher PLSD method) was used to examine differences between any two groups of the four groups.

### Ethics statement

All animal experimental procedures were approved by the Animal Care Committee of Tsurumi University School of Dental Medicine. All experiments using rats were performed in compliance with relevant guidelines and regulations.

## Supplementary Information

Supplementary Information.

## Data Availability

Data that support the findings of this study are available from the corresponding author upon reasonable request.
