# Supplementary material for: Platelet-derived growth factor-BB regenerates functional periodontal ligament in the tooth replantation
Source: Sci Rep. 2022 Feb 25;12:3223. doi: 10.1038/s41598-022-06865-6 (PMC8881622; doi:10.1038/s41598-022-06865-6)
Supplement: Supplementary file 1 — Supplementary Information. [file 41598_2022_6865_MOESM1_ESM.pdf]

Supplementary information

Platelet-derived growth factor-BB regenerates functional periodontal ligament in the tooth replantation

Koichiro Komatsu[1]*, Hisashi Ideno[1], Tatsuya Shibata [1,2], Kazuhisa Nakashima[1], Akira Nifuji[1]

[1]Department of Pharmacology, School of Dental Medicine, Tsurumi University, 2-1-3 Tsurumi, Tsurumi-ku, Yokohama, Japan 230-8501
[2]Division of Dental Pharmacology, Department of Oral Science, School of Dentistry, Oh-u University, 31-1 aza Sankakudoh, Tomita-machi, Kohriyama, Japan 963-8611

page 2 Supplemental Figure S1
page 3 Supplemental Figure S2
page 4 Supplemental Table S1, Table S2
page 5 Supplemental Figure S 3
page 6 Supplemental Table S3, Figure S4
page 7 Supplemental Table S4
page 8 Supplemental Figure S5
page 9 Supplemental Figure S6
page 10 Supplementary Figure S7

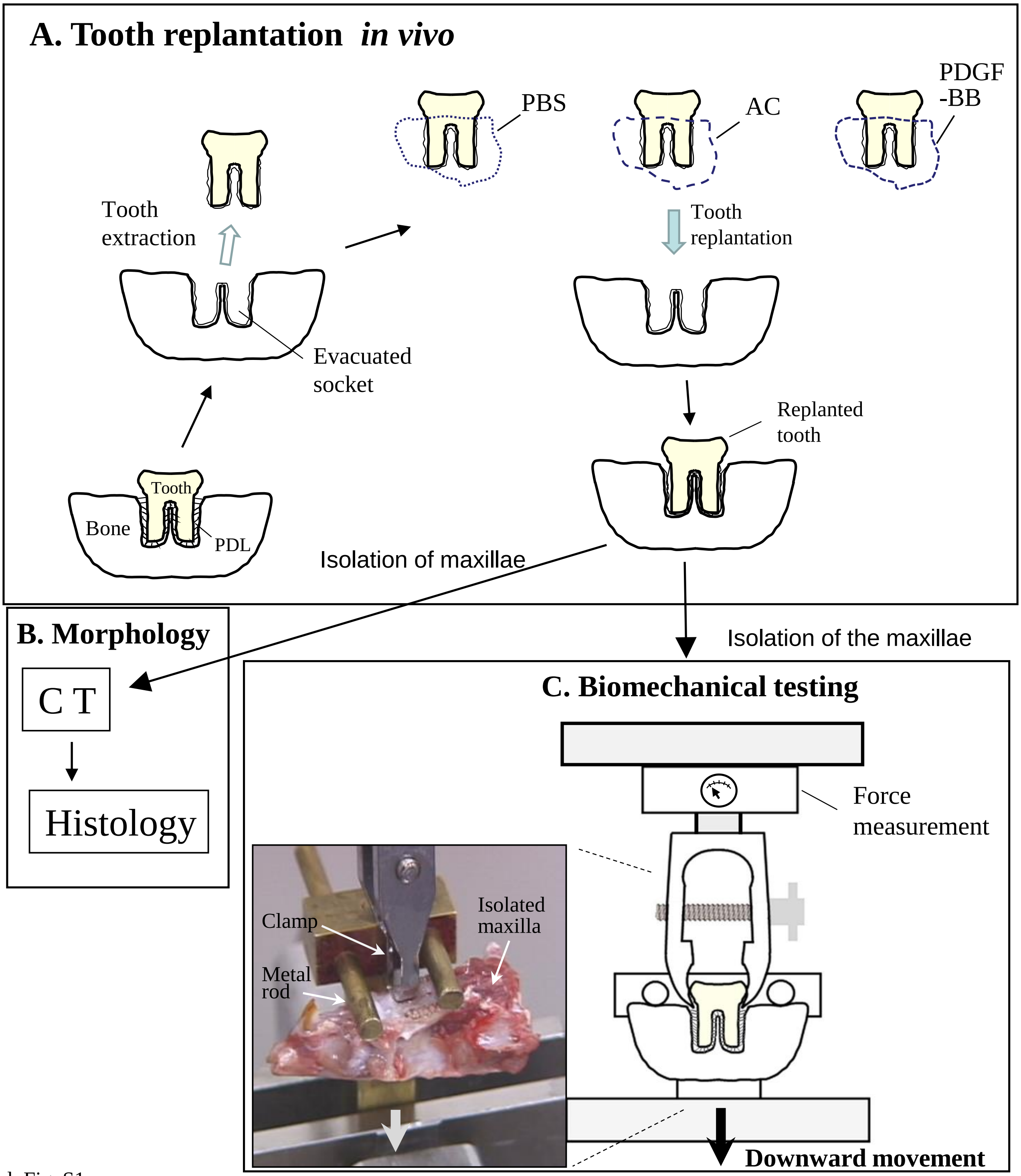

Suppl. Fig. S1.
Diagrammatic representation of the design of the present study analyzing the healing of injured periodontal ligament (PDL) after tooth replantation. (A) Tooth replantation: Rat maxillary first molar teeth were extracted, treated with PBS (PBS group), atelocollagen gel (AC group), or recombinant PDGF-BB (PDGF-BB group) for 5 min, and then replanted into their sockets under anesthesia. (B) Morphology: Replanted teeth were morphologically analyzed by CT and histology 7, 14, and 21 days after replantation. (C) Biomechanical testing: The functional restoration of injured PDL was evaluated by biomechanical testing. Each isolated maxilla containing a replanted tooth is mounted in a materials testing machine. The tooth crown of the replanted tooth is clamped by a metal jaw of tooth clamp connected to a load cell, which is fixed to the *static* upper cross-head, and the isolated maxilla is held by metal rods during testing. By downward movement (black and white arrows) of the lower cross-head, the replanted tooth is gradually extracted from its socket, a continuous load-deformation curve of the healing PDL being recorded by force measurement system. Picture of tooth clamped by jaws of clamp and tooth extraction testing system is also shown.

A

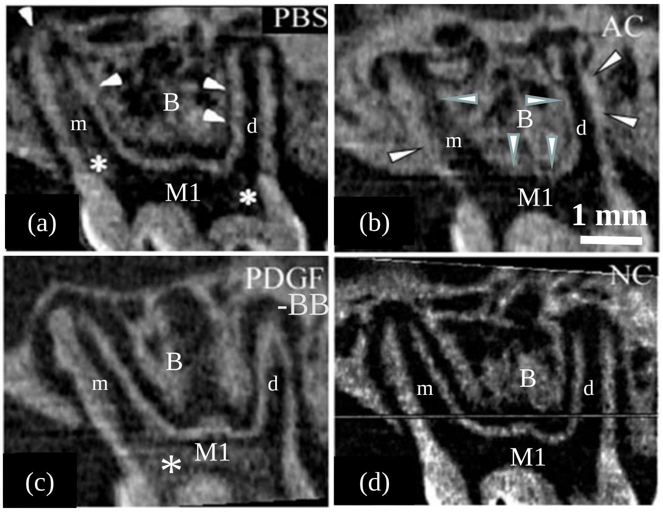

B

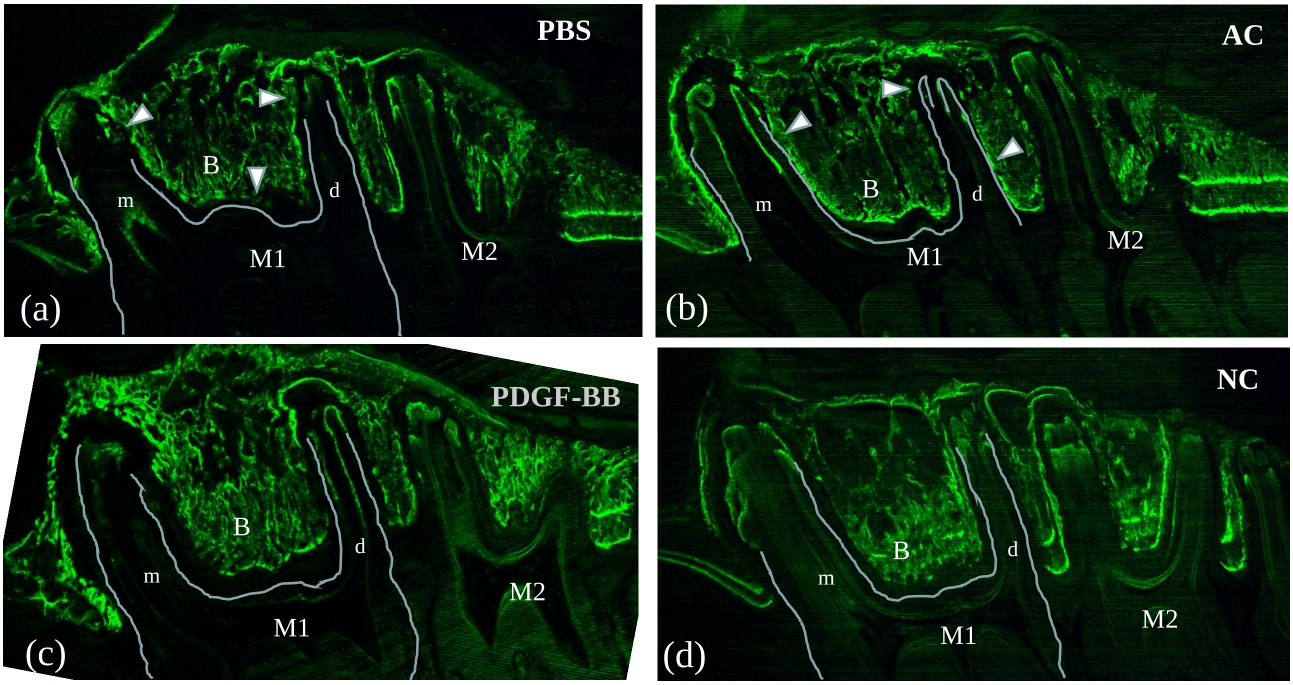

Suppl. Fig. S2.

A. Typical pQ-CT reconstructed images through long axis of mesial and buccal roots of rat maxillary first molars 21 days after replantation. (a) a PBS-pretreated tooth (PBS). (b) an atelocollagen-pretreated tooth (AC). (c) a PDGF-BB-pretreated tooth (PDGF-BB). (d) a non-replanted normal tooth (NC). Arrowheads indicate bony attachments of root surfaces. Asterisks indicate bone-like structures in the dental pulp.

B. Confocal-laser scanning microscopic images show calcein labelings in the replanted and non-replanted teeth. Animals received s.c. injections of calcein on day 0 and day 14 after tooth replantation. Sagittal sections through long axis of mesial and disto-buccal roots of rat maxillary first molars 21 days after replantation. (a) a PBS-pretreated tooth (PBS). (b) an atelocollagen-pretreated tooth (AC). (c) a PDGF-BB-pretreated tooth (PDGF-BB). (d) a non-replanted normal tooth (NC). M1 and M2, maxillary first and second molar teeth; m, mesial root; d, distal root ; B, alveolar bone.

Suppl. Table S1. Number of ankylosed teeth 21 days after tooth replantation evaluated by reconstructed CT images in the four groups.

Days after replantation	Groups			
	NC	PBS	AC	PDGF-BB
21	0 (4)	4 (4)[a, b]	4 (4)[a, b]	1 (4)

Number of rats is shown in parenthesis.
[a] Significant difference from the PDGF-BB group, $p<0.03$ (chi-squared test)
[b] Significant difference from the NC group, $p<0.005$ (chi-squared test)

Suppl. Table S2. Changes in the mean length ± SD (n) of the mesial root of the replanted teeth in the four groups after tooth replantation. The length was measured between the tip of tooth apex and enamel-cement junction along the mesial side of the mesial root on soft X-ray film.

Days	NC	PBS	AC	PDGF
0	1.99 ± 0.15 (9)			
7	2.13 ± 0.13 (10)	1.97 ± 0.14 (9)[a]	1.92 ± 0.09 (9)[b]	2.03 ± 0.07 (8)
14	2.38 ± 0.13 (9)	1.95 ± 0.12 (6)[c]	d	1.95 ± 0.11 (8)[c]
21	2.48 ± 0.14 (10)	d	d	2.05 ± 0.20 (8)[c]

Significant differences from the NC group tested by Scheffé method:
a $p<0.05$; b $p<0.01$; c $p<0.001$.
d Since ankyloses occurred in most of the replanted teeth of the PBS day 21 and in the AC groups day 14 and day 21 (Table 1), we did not measure their lengths of the mesial roots.

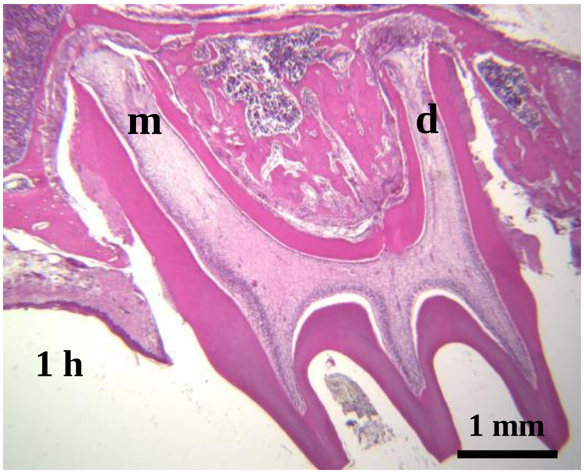

Suppl. Fig. S3. Histologic image of a sagittal section of a rat maxillary first molar 1 h after tooth replantation. Periodontal tissues adhered variously to the tooth and bone surfaces. m, mesial root; d, distal root.

Suppl. Table S3. Measurements of diameters and lengths of tooth roots on horizontal sections and longitudinal sections (below Suppl. Fig. S3) of non-replanted (#5545) and PDGF-pretreated replanted (#5430) maxillary first molar (M1) teeth, and estimation of total surface areas of four roots to calculate a standardized value of mechanical strength (N/mm^2) of PDL of replanted tooth. m1, mesio buccal root; m2, mesio lingual root; d1, disto buccal root 1; d2, disto lingual root .

*Root surface area (mm^2) = Perimeter (mm) × Root length (mm)

**Mean maximum loads are the values presented in Fig. 2d.

		Sectional image				Longitudinal image				
	Tooth roots	Long diameter (mm)	Short diameter (mm)	Shape	Perimeter (mm)	Root length (mm)		Surface area (mm^2)*	Mean max. load (N)**	Mechanical strength (N/mm^2)
#5545 (NC)	m1	0.58	0.36	oval	1.50	2.48		3.72		
	m2	0.31	0.31	circle	0.97	2.30		2.23		
	d1	0.36	0.22	oval	0.92	2.04		1.88		
	d2	0.31	0.31	circle	0.97	2.30		2.23		
							total	10.06	34.1	3.39
#5430 (PDGF-BB)	m1	0.49	0.36	oval	1.34	2.05		2.75		
	m2	0.27	0.27	circle	0.85	1.88		1.57		
	d1	0.31	0.22	oval	0.84	1.80		1.51		
	d2	0.27	0.27	circle	0.85	2.09		1.75		
							total	7.58	18.6	2.45

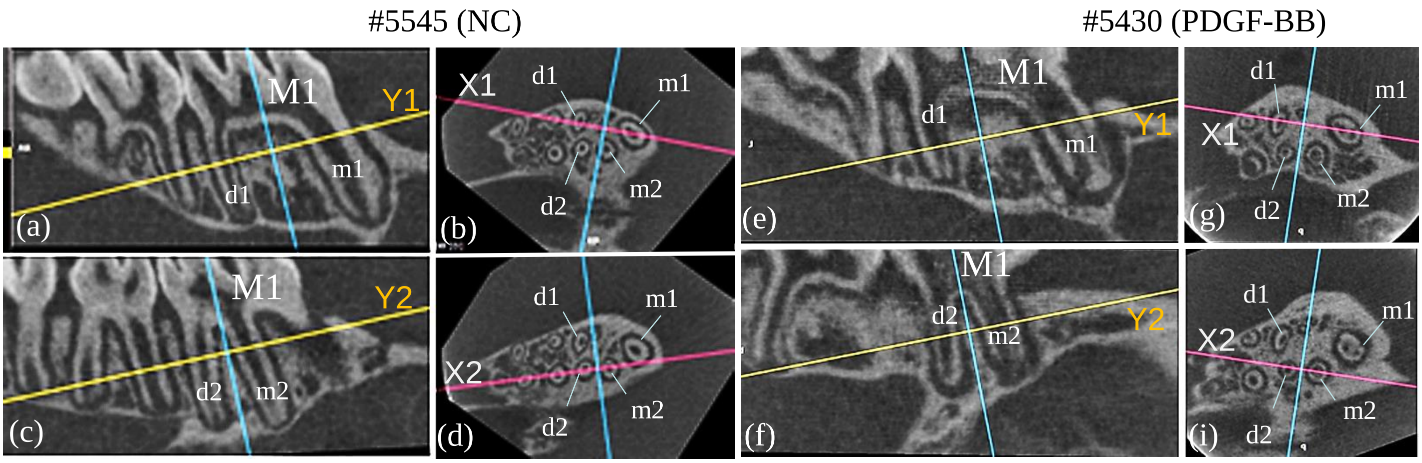

Suppl. Fig. S4. Reconstructed CT images of non-replanted and PDGF-pretreated replanted teeth (M1).
(a, c) Longitudinal sections through X1 (b) and X2 (d) axes, respectively. Non-replanted tooth.
(b, d) Horizontal sections through Y1 (a) and Y2 (c) axes, respectively. Non-replanted tooth.
(e, f) Longitudinal sections through X1 (g) and X2 (i), respectively. PDGF-BB-pretreated replanted tooth.
(g, i) Horizontal sections through Y1 (e) and Y2 (f) axes, respectively. PDGF-BB-pretreated replanted tooth. M1, maxillary first molar; m1, mesio buccal root; m2, mesio lingual root; d1, disto buccal root 1; d2, disto lingual root.

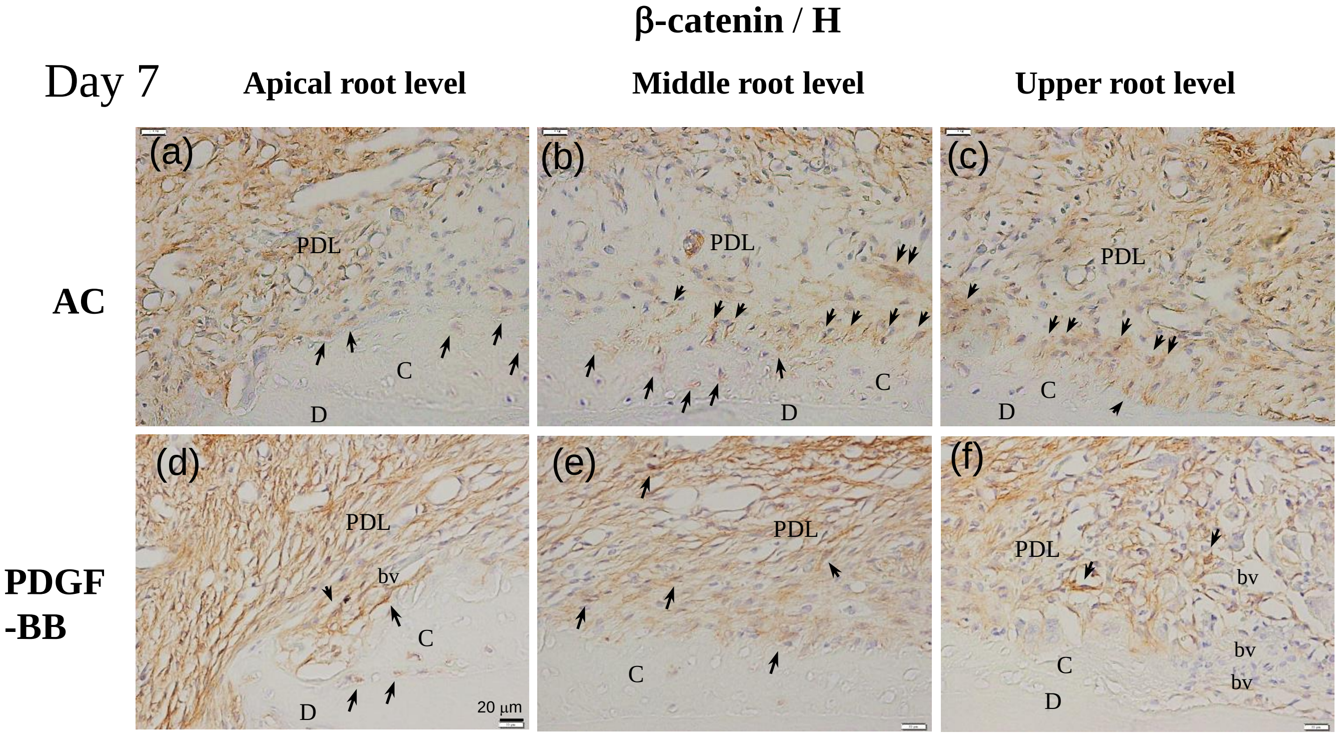

Suppl. Fig. S5.
Immunohistochemistry of β-catenin localization in the periodontal tissues at the apical (a, d), middle (b, e), and upper (c, f) root levels of the replanted teeth of the AC (a-c) and PDGF-BB (d-f) groups day 7. Sections were immunostained with anti-β-catenin primary antibody, and then with peroxidase-conjugated secondary antibody. The sections were treated with DAB substrate, and counterstained with haematoxylin. More β-catenin positive cells (arrows) appear to be observed in the periodontal tissues near the tooth surface of the AC group compared to the PDGF-BB group. PDL, periodontal ligament; C, cementum; D, dentine; bv, blood vessel.

Suppl. Table S4. Primers for RT-PCR

Gene	Forward (5'->3')	Reverse (5'->3')	Reference
GAPDH	GAAGGTCGGAGTCAACGGAT	GATTTTGGAGGGATCTCGCT	BLAST NM_002046.7
CCNB2	CCGACGGTGTCCAGTGATTT	TGTTGTTTTGGTGGGTTGAACT	1
CCND1	GAGAACAAACAGATCATCCGCA	GCTTCGATCTGCTCCTGG	2
CDKN1A	AAGACCATGTGGACCTGTCACTGT	AGGGCTTCCTCTTGGAGAAGATCA	3
CDKN2A	TGCCTTTTCACTGTGTTGGAGTT	TCGCAAGAAATGCCCACAT	3
CTNNB1	TCTGAGGACAAGCCACAAGATTACA	TGGGCACCAATATCAAGTCCAA	4
AXIN2	CTGGCTTTGGTGAACTGTTG	AGTTGCTCACAGCCAAGACA	BLAST NM_001363813.1
RUNX2	TCTTAGAACAAATTCTGCCCTTT	TGCTTTGGTCTTGAAATCACA	5
COL1A1	AGTGGTTTGGATGGTGCCAA	GCACCATCATTTCCACGAGC	BLAST NM_000088.4
ALP	ACAAGCACTCCCACTTCATC	TTCAGCTCGTACTGCATGTC	6

References

1. Wang, X. et al. *OncoTargets and Therapy* 2020:13 6255–6263.

2. Bracken, C. P. et al. *Cancer Research* 2008: 68, 18.

3. Geng, X. et al. *Molecular Therapy* 2021 29, 1279-1293.

4. Gopinathan, G. et al. *Stem Cells Develop* 2019: 28, 5.

5. Kobayashi, E. et al. *BMC Oral Health* 2017:17, 91.

6. Pereira, de Melo D. et al *Front Bioeng Biotech* 2020:23 October 2020.

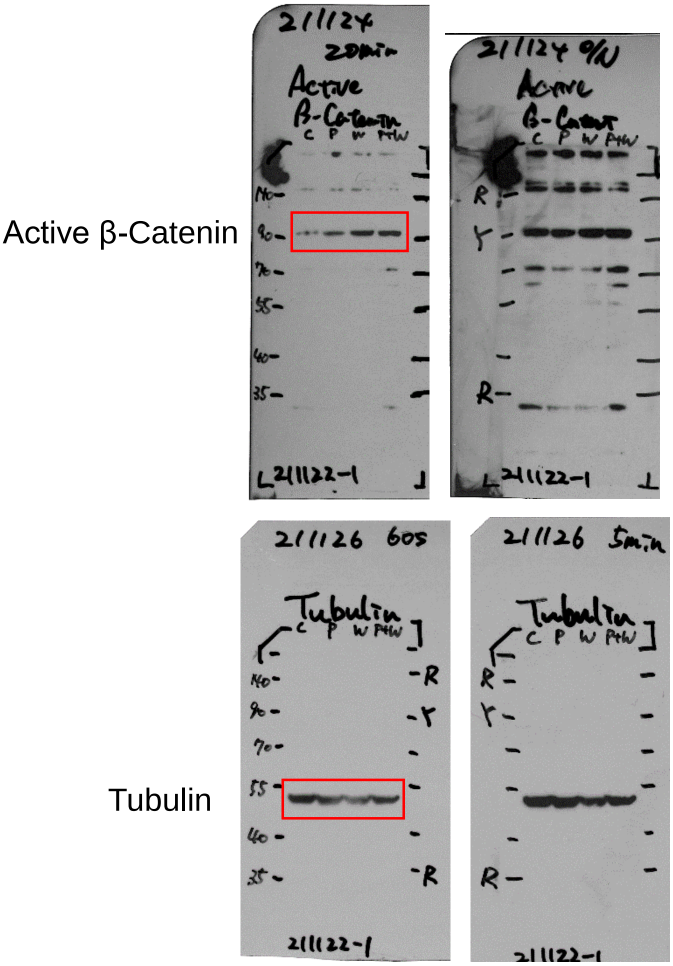

Suppl. Fig. S6. Original blots of Fig. 8B. The following antibodies were used: anti-active β-catenin 1:500 (Millipore, Temecula, CA, USA), anti-Tubulin 1:1500 (Millipore-Sigma, St. Louis, MO, USA).

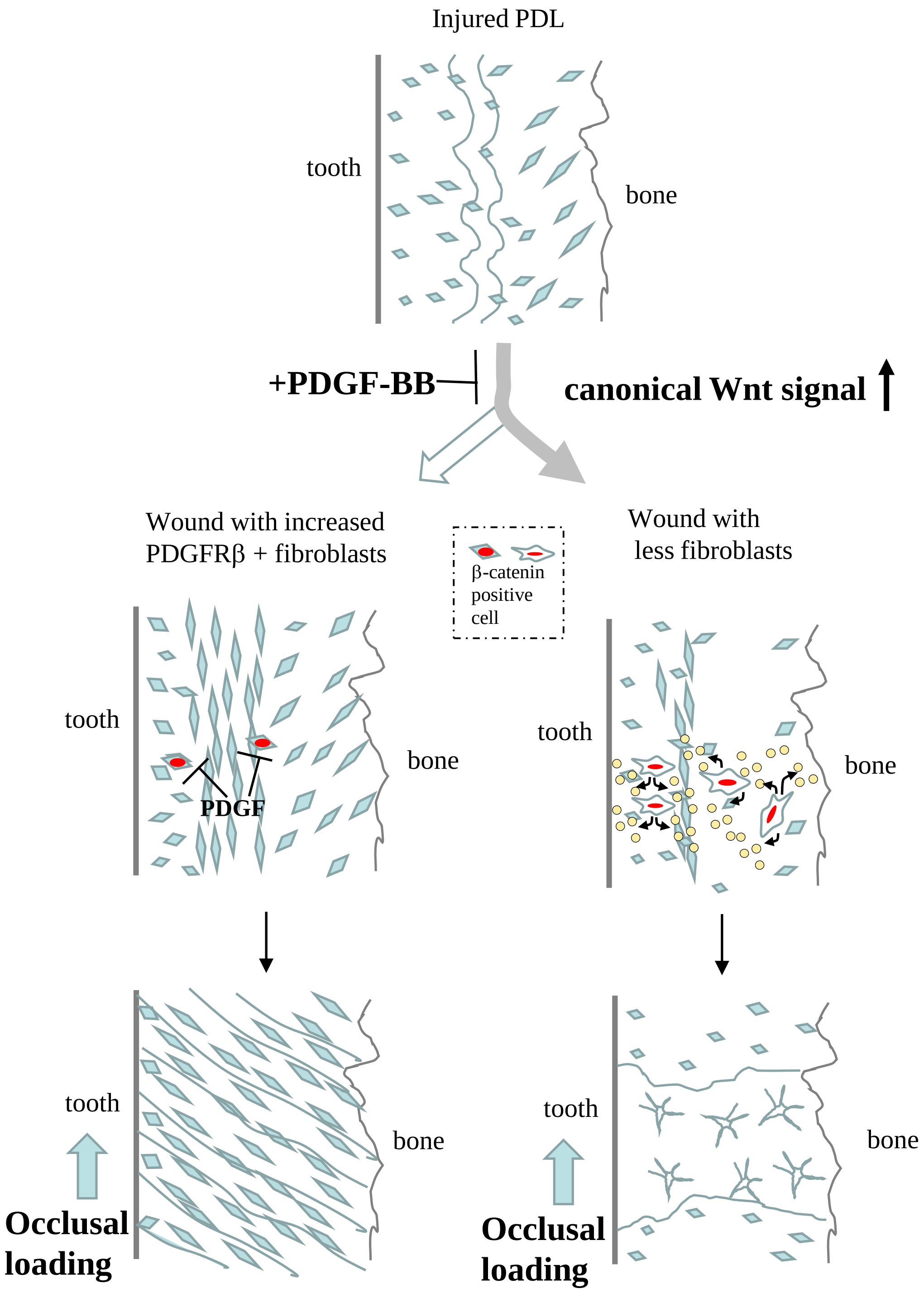

Suppl. Fig. S7.
Diagrammatic representation of effects of PDGF-BB on repair process of the injured PDL after tooth replantation. When the injured PDL is treated with PDGF-BB, PDGF-BB maintains cell viability of PDL tissues and promotes cell migration of PDGF-Rβ positive PDL fibroblasts and inhibits canonical Wnt signal, leading to the healing PDL being subjected to occlusal loading and recovery of the functional PDL structure. Without the growth factor, canonical Wnt signal is upregulated, and ankylotic tissues are formed by osteogenic differentiation from remaining PDL cells or osteogenic cells (β-catenin positive cells).
